# Hepatitis B associated with severe COVID-19: a nationwide cohort study in Sweden

**DOI:** 10.1186/s12985-025-02743-5

**Published:** 2025-04-30

**Authors:** Frida Jakobsson, Osvaldo Fonseca-Rodríguez, Hanna Jerndal, Sebastian Kalucza, Soo Aleman, Marie Eriksson, Anne-Marie Fors Connolly

**Affiliations:** 1 Department of Clinical Microbiology, Umeå University, 90185 Umeå, Sweden; 2 Department of Medicine, Huddinge Karolinska Institutet, 17177 Stockholm, Sweden; 3 Department of Infectious Diseases, Karolinska University Hospital, 141 57 Stockholm, Sweden; 4 Department of Statistics, Economics and Statistics, Umeå School of Business, Umeå University, 901 87 Umeå, SE Sweden

**Keywords:** Viral hepatitis, HBV, Hepatitis B, COVID-19, SARS-CoV2

## Abstract

**Purpose:**

Individuals with severe liver disease are more vulnerable to severe COVID-19, but the association between chronic hepatitis B virus (HBV) infection and severe COVID-19 remains unclear. This study evaluates this relationship.

**Methods:**

We analysed nationwide Swedish data from national databases and healthcare registers, identifying laboratory-confirmed COVID-19 cases from February 2020 to April 2021. Chronic HBV infection was classified into cases with and without cirrhosis. Multivariable logistic regression assessed the association between HBV and severe COVID-19, adjusting for demographics, comorbidities, vaccination, and socioeconomic factors.

**Results:**

Among 1,057,174 COVID-19 cases, 2,902 had chronic HBV infection, which was associated with increased risk of severe COVID-19 (adjusted odds ratio [aOR] 1.242, 95% confidence interval [CI] 1.097–1.403). This risk was significantly higher in HBV individuals with cirrhosis (aOR 2.463, CI 1.546–3.892) compared to those without cirrhosis (aOR 1.183, CI 1.039–1.343). While overall COVID-19 mortality was not significantly elevated in the HBV cohort, patients with cirrhosis showed a higher, though nonsignificant, mortality risk (aOR 2.350, CI 0.921–5.203).

**Conclusion:**

This nationwide study highlights an increased risk of severe COVID-19 in individuals with chronic HBV, particularly those with cirrhosis. Geographic and socioeconomic factors further influence outcomes. These findings underscore the need to consider HBV status in COVID-19 risk assessments. Future studies should explore these associations in the context of evolving SARS-CoV-2 variants and widespread vaccination.

**Supplementary Information:**

The online version contains supplementary material available at 10.1186/s12985-025-02743-5.

## Background

Emerging and re-emerging viral infections continue to pose significant challenges to global health, particularly within healthcare environments. In recent years, multiple outbreaks of zoonotic viruses have been documented, highlighting the persistent threat these pathogens represent [1–5]. Severe acute respiratory syndrome coronavirus 2 (SARS-CoV-2) that causes the coronavirus disease 2019 (COVID-19) hit the entire world and was declared as a global pandemic in March 2020, and soon became the most important concern for all patients with chronic diseases. Some people experience an asymptomatic or mild infection, while others can face serious and possibly even life-threatening symptoms and complications, commonly referred to as severe COVID-19. The United States Centers for Disease Control and Prevention (CDC) defines severe outcomes of COVID-19 as hospitalization, admission to the intensive care unit (ICU), intubation or mechanical ventilation, or death [6]. Globally, COVID-19 has caused over seven million deaths by March 2025 [7]. SARS-CoV-2 causes a wide range of symptoms and multiple other organ systems are affected. There are well documented short- and long-term effects on gastrointestinal and hepatic systems [8, 9]. Notably liver injury is a commonly observed complication in COVID-19 patients and is associated with unfavourable clinical outcomes [10, 11]. It is also described previously that patients with liver cirrhosis have a higher risk for severe COVID-19[12, 13]. Liver cirrhosis is caused by chronic liver damage that leads to scarring, where liver cells are replaced by collagen (fibrosis). The extent of fibrosis is graded in four different stages. Liver decompensation refers to the stage of cirrhosis when the liver can no longer maintain normal function, leading to complications such as ascites, jaundice, variceal bleeding, or hepatic encephalopathy.

Viral hepatitis is the main cause of liver cirrhosis, liver failure, and hepatocellular carcinoma (HCC) globally(14). Chronic hepatitis B virus (HBV) infection is a global health burden with substantial morbidity, mortality, and health cost. It is estimated that approximately 257.5 million people worldwide have a chronic HBV infection(15) with a significant regional variation in HBV prevalence [16]. Most patients with chronic HBV-infection in Sweden are infected during their childhood, in their previous home country, before arrival to Sweden(17).

Both SARS-CoV-2 and HBV can cause liver damage and given the immune dysregulation that accompanies chronic inflammation in the liver, it has been hypothesized that patients with chronic HBV-infection may be particularly vulnerable to severe COVID-19. However, previous studies are inconsistent regarding whether HBV-infection increases the risk of severe COVID-19[18–20]. Two independent meta-analyses from 2023 showed that there seems to be an association between HBV and severe COVID-19 but emphasized the need for further studies with more global data to establish a clearer understanding[21, 22]. Thus, at a nationwide population level, where every individual in the country that has a laboratory-verified positive SARS-CoV-2 test is included, the risk for severe COVID-19 in patients with chronic HBV-infection is unknown.

The primary objective of our study was to determine the risk of severe COVID-19 in individuals with chronic HBV infection compared to those without HBV. Additionally, we aimed to determine and compare the risk in hepatitis B individuals with or without cirrhosis diagnosis.

## Methods

### Source of data and study population

In this nationwide register-based cohort study we used data from Swedish national public authority registers and quality registers. In Sweden, since February 2020, COVID-19 has been a notifiable disease, and all laboratory-verified SARS-CoV-2 infections are reported to the communicable disease surveillance system, SmiNet, at the Swedish Public Health Agency. In our study the personal identification numbers for all individuals with a laboratory verified SARS-CoV2 infection, between 1 February 2020 and 25 April 2021, were used to cross link data from Statistics Sweden to obtain data on country of birth and socioeconomic data from Longitudinal Integrated Database for Health Insurance and Labor Market Studies (LISA) registry. This data was also linked to the Inpatient Registry (1987-2021), Outpatient Registry (1997-2021) and Cause of Death Registry (2020-21) from the Swedish National Board of Health and Welfare, and to the Swedish Intensive Care Registry (2020-21). All data were pseudonymised by Statistics Sweden and the National Board of Health and Welfare.

To identify the closest date of infection with SARS-CoV-2 from the registry database, we used the earliest of following reported dates: date of disease onset, sample date, diagnosis date, and date of report to SmiNet. Only first infections were included. We excluded individuals with less than 30 days follow-up and individuals <18 years old.

### Chronic hepatitis B virus infection

Individuals were considered to have chronic hepatitis B virus (HBV) infection if they had received a diagnosis for chronic HBV infection prior to COVID-19 index date. The identification of these individuals was based on International Statistical classification of Diseases (ICD). The ICD-10, and ICD-9 diagnosis codes, B181 respectively 070D were obtained from both the inpatient register (1987-2021) and outpatient registers (1997-2021). The HBV infected individuals were further categorized into two groups: 1) Hepatitis B without cirrhosis and 2) Hepatitis B with cirrhosis, with or without liver decompensation. Compensated cirrhosis is diagnosed through liver biopsy or elastography. Decompensated cirrhosis is diagnosed based on symptoms, radiological findings, clinical parameters, and laboratory tests. The diagnosis codes for cirrhosis and liver decompensation were obtained from the inpatient and outpatient register. (Table 1, Additional files). Cirrhosis was defined as diagnosis given at any time before COVID-19 index date and liver decompensation diagnosis given at least 2 weeks before COVID-19 index date.

**Table 1:** Baseline characteristics of the study population

	**COVID-19 without HBV** **n = 847 822**	**COVID-19 and HBV** **N=2902**	**COVID-19 and HBV without cirrhosis** **n = 2805**	**COVID-19 and HBV with cirrhosis** **n = 97**
Age (years) Mean (SD)	44.3 (17.2)	46.2 (13.3)	45.7 (13.1)	58.6 (12.0)
**Sex, No (%)**				
Male	410 282 (48.4%)	1602 (54.9%)	1532 (54.7%)	70 (72.2%)
Female	437 540 (51.6%)	1313 (45.1%)	1286 (45.3%)	27 (27.8%)
**Vaccination prior to COVID-19, No (%)**				
No	1 042 055 (98.9%)	Too few	2 775 (98.9%)	Too few
Yes	9 179 (1.1%)	Too few	30 (1.1%)	Too few
**Region of birth, no (%)**				
Northern Europe	655 207 (77.3%)	326 (11.2%)	304 (10.8%)	22 (22.7%)
Rest of Europe	37 301 (4.4%)	267 (9.2%)	257 (9.1%)	10 (10.3%)
Asia	99 934 (11.8%)	1 401 (48.3%)	1 364 (48.5%)	37 (38.1%)
Africa	16 732 (2.0%)	499 (17.2%)	481 (17.2%)	18 (18.6%)
America	11 223 (1.2%)	12 (0.4%)	10 (0.3%)	2 (2.1%)
Other/missing	27 425 (3.2%)	397 (13.7%)	389 (13.9%)	8 (8.2%)
**wCCI (excl liver disease) No (%)**				
0	792 838 (93.5%)	2392 (82.4%)	2334 (83.2%)	58 (59.8%)
1–2	30 468 (3.6%)	339 (11.7%)	333 (11.9%)	5 (5.5%)
3–4	8 108 (1.0%)	62 (2.1%)	55 (2.0%)	7 (7.2%)
>=5	16 408 (1.9%)	109 (3.8%)	82 (2.9%)	27 (27.8%)
**Level of education, No (%)**				
<9 years	133 270 (15.7%)	870 (30.0%)	829 (29.6%)	41 (42.3%)
10–12 years	369 765 (43.6%)	1 153 (39.7%)	1 122 (40.0%)	31 (32.0%)
>12 years	325 070 (38.3%)	733 (25.3%)	715 (25.5%)	18 (18.6%)
Missing	19 717 (2.3%)	146 (5.0%)	139 (5.0%)	7 (7.2%)
**Income, No (%)**				
Lowest	140 840 (16.6%)	1 099 (37.9%)	1 047 (37.3%)	52 (53.6%)
Low	169 976 (20.0%)	715 (24.%)	698 (24.9%)	17 (17.5%)
Middle	174 817 (20.6%)	495 (17.1%)	485 (17.3%)	10 (10.3%)
High	176 063 (20.8%)	321 (11.1%)	311 (11.1%)	10 (10.3%)
Highest	175 478 (20.7%)	215 (7.4%)	207 (7.4%)	8 (8.2%)
Missing	10 648 (1.3%)	57 (2.0%)	57 (2.0%)	0

Data are n (%) or mean (SD). Percentages are calculated as a proportion of the included study population. SD= standard deviation. OR=odds ratio. wCCI=weighted Charlson Comorbidity Index, excluding liver disease. Income is divided in quintiles where quintile 1 is the highest income, quintile 5 is the lowest income. HBV= chronic Hepatitis B virus infection

### Variables and definitions

Covariates included sex, age (at index date), SARS-CoV-2 vaccination status (at least two weeks before index date), co-morbidities, geographic region of birth and socioeconomic factors (income and education level). The weighted Charlson Comorbidity Index (wCCI) [23] was calculated to consider the burden of comorbidities, based on historical registry data from the outpatient register, inpatient register, and the cancer register using a method specifically adapted to Swedish National Registers [24]. To ensure complications due to COVID-19 were not included in the wCCI, the calculation was stopped one month prior to the index date. If there were no wCCI diagnosis codes in the outpatient register, inpatient register, or the cancer register, the individual was given a wCCI of zero. Since our main objective was viral hepatitis, we removed liver disease from the wCCI categories. The country of birth was defined as the birth country registered for that specific individual in the Total Population Registry at Statistics Sweden. The birth countries were divided in larger regions, according to international standards based on ISO 3166. The regions were Northern Europe, other Europe, Africa, Asia, America or other (includes other regions or missing data) (Table 6, Additional files). The socioeconomic variables: income and education, were received from the LISA registry. Income was defined as the annual disposable income divided into quintiles, where quintile one is the highest income, quintile five is the lowest income. Education was divided in three stages: primary, secondary, and tertiary education. If a variable was not registered, it was grouped in category named “missing”.

The primary outcome of severe COVID-19 was based on a composite measure including hospitalization, need for intensive care unit (ICU) treatment, and death by COVID-19. Hospitalization, need for ICU care or death due to COVID-19 was defined through the ICD10 codes U071 or U072 as main or contributing diagnoses within one month post index date in the inpatient-, intensive care- or cause of death registries, respectively. Secondary outcome included death due to COVID-19, and all-cause mortality within 30 days post index date.

### Statistical analysis

Univariable and multivariable logistic regression was used to determine whether patients with chronic HBV had increased risk of severe COVID-19. The multivariable analyses included the potential confounders: age (years), sex, region of birth, COVID-19 vaccination (yes/no), comorbidity burden measured by wCCI excluding liver disease (0, 1–2, 3–4, or >=5), education and income. Outcome was presented by odds ratios (OR) with 95% confidence intervals (CI). Confidence intervals and p-values were not adjusted for multiple testing. Missing data were handled by adding these as a separate category. Statistical analyses were carried out using R 4.x.x (R Core Team, 2021).

## Results

### Baseline characteristics of the COVID-19 cohort

**Fig. 1:**
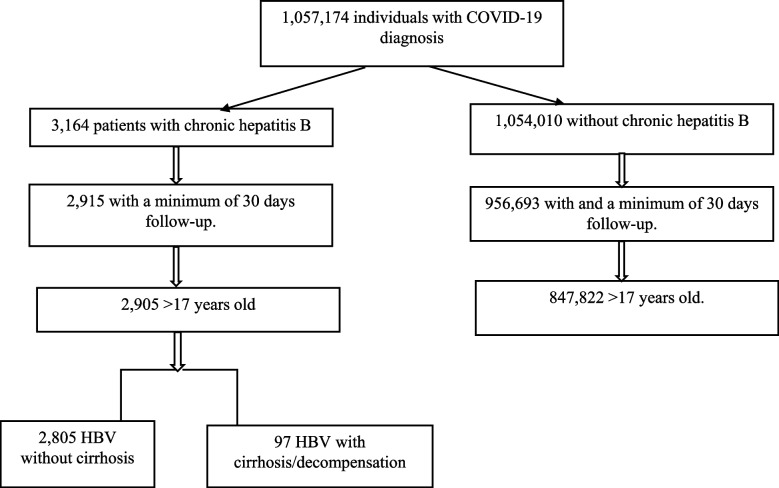
Flowchart over individuals included

In this study we had data for 850 724 individuals with COVID-19 (from February 2020 to 25 April 2021), with at least 1 month follow up, and who were 18 years and older. There were 2902 COVID-19 patients with a chronic HBV diagnosis, and out of these, 97 individuals had cirrhosis (Fig. 1).

The average age was 44.3 (standard deviation [SD] 17.2) years in the COVID-19 cohort, vs 45.7 (SD 13.1) years in the HBV-cohort without cirrhosis and 58.6 (SD 12.0) years in HBV-infected patients with cirrhosis, respectively. Most individuals without hepatitis B were born in Northern Europe (77.3%), whereas most individuals with HBV infection were born in Asia 1401 (48.3%) or Africa 499 (17.2%). The HBV cohort had lower socioeconomic status with 1099 individuals (37.9%) in the group of lowest income quartile and 870 (30.0%) with <9 years of education (Table 1).

### Chronic HBV infection associates with severe COVID-19

In the univariable analyses of the primary outcome (hospitalization/ICU/death due to COVID-19), chronic HBV infection associated with severe COVID-19 (OR 1.954, CI: 1.746–2.179). That remained significant even after adjustment for potential confounders (aOR 1.242, CI 1.097–1.403) in the multivariate analysis. Higher age, male sex, higher wCCI, lower socioeconomic status and all regions of birth compared to Northern Europe associated with severe COVID-19. Individuals born in Africa had the highest risk for hospitalization/ICU/death compared to individuals from other regions, (aOR 2.549, CI 2.402–2.703) (Table 2).

**Table 2:** Primary outcome. Severe COVID-19. Hospitalization/ICU/death by COVID-19 combined vs non-hospitalized

	**Dependent: Severity**	**Non-hospitalized, No (%)**	**Hospitalized/ICU/Dead No (%)**	**OR (univariable)**	**OR (multivariable)**
Hepatitis B	No hepatitis B	790349 (93.2)	57473 (6.8)	-	-
	Hepatitis B	2541 (87.6)	361 (12.4)	1.954 (1.746–2.179, p<0.001)	1.242 (1.097–1.403, p=0.001)
Age	Mean (SD)	42.7 (15.9)	66.6 (18.0)	1.080 (1.079–1.080, p<0.001)	1.077 (1.076–1.077, p<0.001)
Sex	Women	413758 (94.3)	25091 (5.7)	-	-
	Men	379132 (92.1)	32743 (7.9)	1.424 (1.400–1.449, p<0.001)	1.784 (1.750–1.820, p<0.001)
Region of birth	Northern Europe	614466 (93.7)	41067 (6.3)	-	-
	Other Europe	34682 (92.3)	2886 (7.7)	1.245 (1.197–1.295, p<0.001)	1.454 (1.390–1.521, p<0.001)
	Africa	15618 (90.6)	1613 (9.4)	1.545 (1.466–1.628, p<0.001)	2.549 (2.402–2.703, p<0.001)
	Asia	92428 (91.2)	8907 (8.8)	1.442 (1.408–1.477, p<0.001)	2.086 (2.026–2.148, p<0.001)
	America	10191 (90.7)	1044 (9.3)	1.533 (1.436–1.634, p<0.001)	2.093 (1.949–2.245, p<0.001)
	Other/missing	25505 (91.7)	2317 (8.3)	1.359 (1.301–1.420, p<0.001)	1.749 (1.665–1.837, p<0.001)
COVID-19 vaccination	0	784573 (93.2)	56941 (6.8)	-	-
	1	8317 (90.3)	893 (9.7)	1.479 (1.379–1.585, p<0.001)	0.560 (0.518–0.606, p<0.001)
wCCI group (excl liver disease)	0	752170 (94.5)	43960 (5.5)	-	-
	1–2	24044 (79.6)	6162 (20.4)	4.385 (4.257–4.516, p<0.001)	2.374 (2.290–2.461, p<0.001)
	3–4	4687 (58.6)	3315 (41.4)	12.102 (11.563–12.664, p<0.001)	2.604 (2.464–2.753, p<0.001)
	>=5	11989 (73.2)	4397 (26.8)	6.275 (6.054–6.504, p<0.001)	2.904 (2.775–3.038, p<0.001)
Education	Tertiary	311056 (95.5)	14747 (4.5)	-	-
	Secondary	347544 (93.7)	23374 (6.3)	1.419 (1.389–1.449, p<0.001)	1.150 (1.124–1.178, p<0.001)
	Primary	116476 (86.8)	17664 (13.2)	3.199 (3.126–3.273, p<0.001)	1.315 (1.279–1.352, p<0.001)
	Missing	17814 (89.7)	2049 (10.3)	2.426 (2.310–2.546, p<0.001)	1.519 (1.421–1.623, p<0.001)
Income	Highest	167181 (95.2)	8512 (4.8)	-	-
	High	168963 (95.8)	7421 (4.2)	0.863 (0.836–0.891, p<0.001)	1.092 (1.056–1.129, p<0.001)
	Middle	166740 (95.1)	8572 (4.9)	1.010 (0.979–1.041, p=0.538)	1.292 (1.250–1.336, p<0.001)
	Low	154795 (90.7)	15896 (9.3)	2.017 (1.963–2.073, p<0.001)	1.614 (1.565–1.665, p<0.001)
	Lowest	124968 (88.0)	16971 (12.0)	2.667 (2.596–2.740, p<0.001)	1.904 (1.842–1.967, p<0.001)
	Missing	10243 (95.7)	462 (4.3)	0.886 (0.804–0.974, p=0.013)	1.540 (1.377–1.718, p<0.001)

Univariable and multivariable (adjusting for all other factors in the column) logistic regression modelling severe COVID-19 (Hospitalization/ICU/death). Exposure (Hepatitis B) in two categories (yes/no). Frequency (%), and odds ratios (OR) with 95% confidence intervals. OR=odds ratio. ICU= intensive care unit admission. wCCI=weighted Charlson Comorbidity Index, excluding liver disease. Income is divided in quintiles, where quintile 1 is the highest income, quintile 5 is the lowest income

**Fig. 2:**
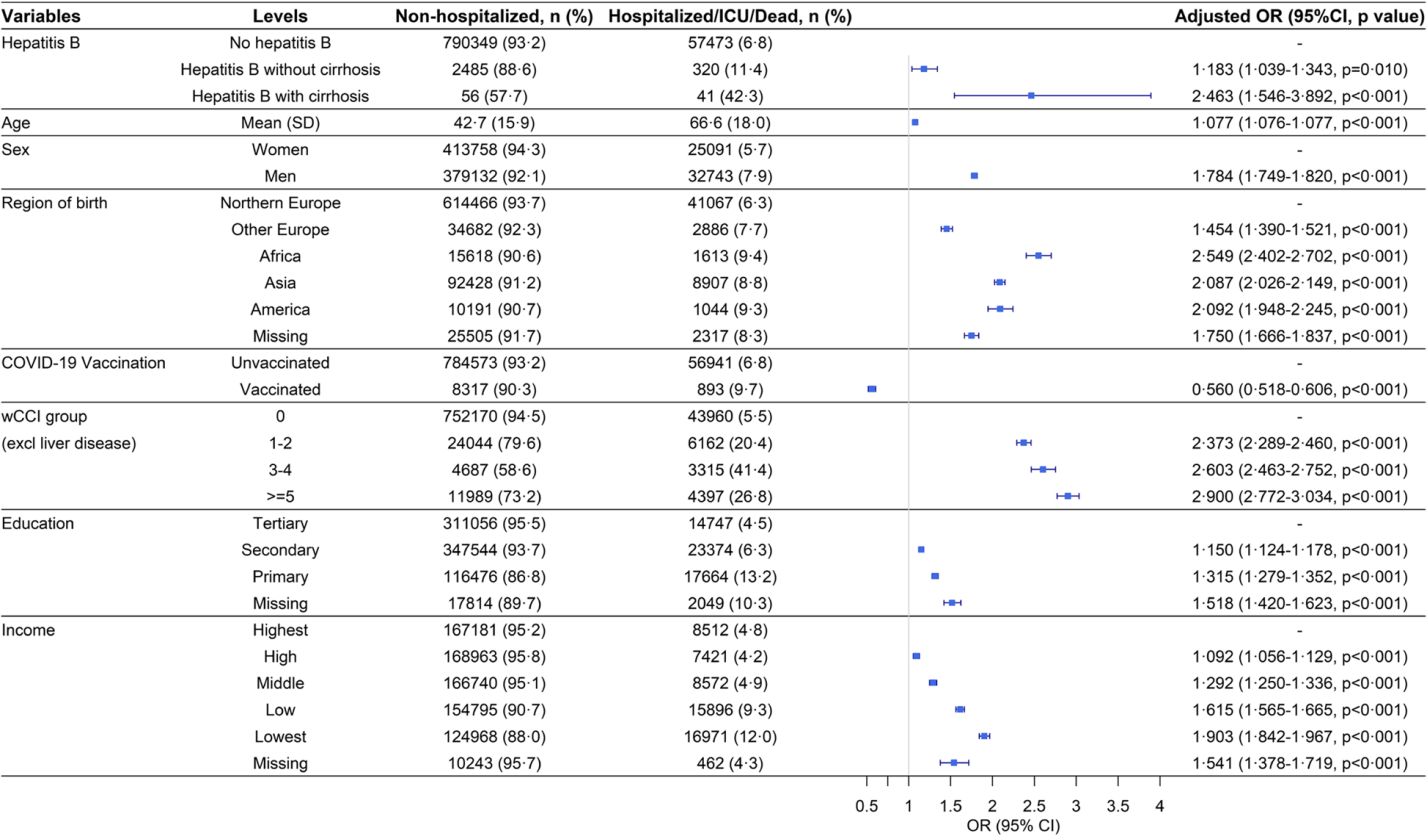
Primary outcome: Severe COVID-19. Hepatitis B with and without cirrhosis. Hospitalization/ICU/death by COVID-19 combined vs. non-hospitalized. Univariable and multivariable (adjusting for all other factors in the column) logistic regression modelling severe Covid-19 (Hospitalization/ICU/death). Exposure (Hepatitis B) in three categories (No hepatitis B, Hepatitis B without cirrhosis, and Hepatitis B with cirrhosis). Frequency (%), and odds ratios (OR) with 95% confidence intervals

COVID-19 vaccination was protective against severe COVID-19 in the multivariate analysis (aOR 0.560, CI 0.518–0.606). Compared to COVID-19 patients without HBV, an increased risk of severe COVID-19 was still present in HBV patients without liver cirrhosis (aOR 1.183, CI 1.039–1.343), and further elevated in HBV infected persons with liver cirrhosis (aOR 2.463, CI 1.546–3.892) (Fig. 2).

### HBV and risk of death due to COVID-19

The overall result of the secondary outcome in this study, death due to COVID-19, shows a similar pattern as that of the primary composite outcome. However, there were fewer events, and the observed increased risk of death by COVID-19 in chronic HBV infected persons was not statistically significant compared to those without HBV infection (aOR 1.168, CI 0.765–1.715) in the multivariable analysis (Table 2, Additional files). Patients with HBV infection and liver cirrhosis had a higher risk for death due to COVID-19 in the univariable analyse (OR 5.641 CI 2.372–11.310), but in the multivariable analysis it was not statistically significant (aOR 2.350 CI 0.921–5.203 p=0.050)) (Table 3).

**Table 3:** Secondary outcome. Death by COVID-19. Hepatitis B with or without cirrhosis. Death by covid vs non-hospitalized/hospitalized/ICU combined

**Dependent: Severity Death**		**Non-hosp./Hosp/ICU**	**Dead**	**OR (univariable)**	**OR (multivariable)**
**Hepatitis B**	No hepatitis B	836292 (98.6)	11530 (1.4)	-	-
	Hepatitis B without cirrhosis	2783 (99.2)	22 (0.8)	0.573 (0.365–0.849, p=0.009)	1.003 (0.616–1.548, p=0.990)
	Hepatitis B with cirrhosis	90 (92.8)	7 (7.2)	5.641 (2.372–11.310, p<0.001)	2.350 (0.921–5.203, p=0.050)
**Age**	Mean (SD)	43.8 (16.6)	83.1 (9.9)	1.147 (1.145–1.149, p<0.001)	1.133 (1.131–1.136, p<0.001)
**Sex**	Women	433583 (98.8)	5266 (1.2)	-	-
	Men	405582 (98.5)	6293 (1.5)	1.278 (1.231–1.326, p<0.001)	2.353 (2.250–2.461, p<0.001)
**Region of birth**	Northern Europe	645459 (98.5)	10074 (1.5)	-	-
	Other Europe	37119 (98.8)	449 (1.2)	0.775 (0.704–0.851, p<0.001)	0.956 (0.856–1.066, p=0.424)
	Africa	17121 (99.4)	110 (0.6)	0.412 (0.339–0.494, p<0.001)	1.336 (1.075–1.642, p=0.007)
	Asia	100738 (99.4)	597 (0.6)	0.380 (0.349–0.412, p<0.001)	0.851 (0.768–0.942, p=0.002)
	America	11154 (99.3)	81 (0.7)	0.465 (0.371–0.575, p<0.001)	0.966 (0.751–1.224, p=0.779)
	Other/missing	27574 (99.1)	248 (0.9)	0.576 (0.506–0.652, p<0.001)	1.041 (0.901–1.198, p=0.579)
**COVID-19 Vaccination**	0	830242 (98.7)	11272 (1.3)	-	-
	1	8923 (96.9)	287 (3.1)	2.369 (2.099–2.663, p<0.001)	0.598 (0.524–0.681, p<0.001)
**wCCI group (excl liver disease)**	0	789545 (99.2)	6585 (0.8)	-	-
	1–2	28458 (94.2)	1748 (5.8)	7.365 (6.975–7.772, p<0.001)	2.022 (1.896–2.156, p<0.001)
	3–4	6462 (80.8)	1540 (19.2)	28.574 (26.886–30.353, p<0.001)	2.784 (2.589–2.992, p<0.001)
	>=5	14700 (89.7)	1686 (10.3)	13.752 (13.000–14.539, p<0.001)	3.008 (2.809–3.221, p<0.001)
**Education**	Tertiary	323916 (99.4)	1887 (0.6)	-	-
	Secondary	366704 (98.9)	4214 (1.1)	1.973 (1.868–2.083, p<0.001)	1.199 (1.128–1.276, p<0.001)
	Primary	129108 (96.2)	5032 (3.8)	6.690 (6.344–7.058, p<0.001)	1.252 (1.175–1.335, p<0.001)
	Missing	19437 (97.9)	426 (2.1)	3.762 (3.379–4.179, p<0.001)	1.641 (1.426–1.885, p<0.001)
**Income**	Highest	174801 (99.5)	892 (0.5)	-	-
	High	175704 (99.6)	680 (0.4)	0.758 (0.686–0.838, p<0.001)	1.089 (0.979–1.209, p=0.115)
	Middle	174092 (99.3)	1220 (0.7)	1.373 (1.260–1.498, p<0.001)	1.480 (1.348–1.625, p<0.001)
	Low	166233 (97.4)	4458 (2.6)	5.255 (4.892–5.652, p<0.001)	2.035 (1.877–2.209, p<0.001)
	Lowest	137646 (97.0)	4293 (3.0)	6.112 (5.688–6.575, p<0.001)	2.372 (2.178–2.585, p<0.001)
	Missing	10689 (99.9)	16 (0.1)	0.293 (0.171–0.464, p<0.001)	1.333 (0.757–2.184, p=0.285)

Univariable and multivariable (adjusting for all other factors in the column) logistic regression modelling severe COVID-19 (Death). Exposure (Hepatitis B) in three categories (No hepatitis B, hepatitis B without cirrhosis, and hepatitis B with cirrhosis). Frequency (%), and odds ratios (OR) with 95% confidence intervals. ICU: intensive care unit admission. Non-hosp: Non-hospitalised. Hosp: Hospitalised. wCCI: weighted Charlson Comorbidity index

### HBV and all-cause mortality following COVID-19

When analysing the risk for death by any cause 30 days after COVID-19, HBV infection did not associate with a significantly increased risk compared to persons without HBV infection (aOR 1.100 CI 0.735–1.589) (Table 3, Supplementary). Patients with HBV infection and liver cirrhosis however, seemed to have a higher all-cause mortality, but this difference did not reach statistical significance in the multivariable analysis (aOR 2.354, CI 0.975–5.038) (Table 4. Additional files).

## Discussion

In this nationwide, population-based cohort of COVID-19 patients, we found an increased risk of severe COVID-19 (hospitalization, ICU treatment or death) in individuals with chronic HBV infection, even after adjusting for potential confounders. In addition to an increased risk in individuals who were male, elderly, non-vaccinated, and with low- socioeconomic status (SES), the multivariable models also showed that individuals diagnosed with HBV and born in Africa had a higher risk of severe COVID-19, including an increased risk of death due to COVID-19, compared to individuals with HBV infection born in other regions.

A potential mechanism, that could explain that persons with chronic hepatitis B with- or without cirrhosis have higher risk for severe COVID-19, is T-cell exhaustion. Chronic HBV leads to persistent antigen exposure, causing exhaustion of virus-specific CD8+ T cells. T-cell exhaustion is characterised by poor effector function, diminished proliferation, and differentiation, decreased cytokine responses, and high expression of inhibitory receptors which results in reduced capacity to control not only HBV but also other viral infections [25]. Chronic HBV infection also affects NK cells and dendritic cells, and the impact of chronic HBV infection on the immune system would therefore result in diminished immune function, with increased risk for severe disease [26, 27]. In addition, HBV patients with advanced cirrhosis, and decompensation have impaired immune regulation, placing these individuals at further risk for severe COVID-19 [28, 29]. SARS-CoV-2 infection itself contributes to liver injury, and the etiological mechanisms include severe inflammatory response, anoxia, drug-induced liver injury, direct cytotoxicity, as well as reactivation of pre-existing liver disease [30, 31]. All together, the findings in our study could be caused by the described mechanisms.

A potential explanation of our finding that individuals born in other regions than northern Europe, and especially those born in African countries, had higher risk for severe COVID-19 could be that individuals that moved to Sweden more recently lack register information on history of e.g. hospital admissions. Hence, wCCI may not fully capture and adjust for comorbidity in this group, and their higher risk may in part be caused by residual confounding. However, according to a report from the Public Health Agency of Sweden, individuals born in other countries, particularly in Africa and the Middle East, exhibited a greater risk of ICU admissions and higher mortality rates compared to those born in Sweden during the period spanning 2020-2021[32]. Similar patterns have been discerned in other Western nations(33, 34). This indicates that individuals that have migrated to Sweden, especially from African regions, might be more vulnerable to severe COVID-19.

Our study has several strengths. Our study includes all known laboratory-verified COVID-19 cases in Sweden, and thereby the proportion of included known cases is complete. Our study has a larger sample size compared to prior investigations which enhances the statistical power and reliability of our findings(18, 20, 35). By leveraging the comprehensive, nationwide data of the Swedish healthcare registers, our study cohort incorporated over 1,000,000 individuals in total, representative of the entire COVID-19 spectrum. This expanded dataset facilitated the inclusion of a diverse array of variables in our analytical model, permitting a more meticulous adjustment for potential confounding factors than was feasible in earlier studies. We also included all COVID-19 patients diagnosed with HBV on a nationwide scale. Many other studies focused mainly on hospitalized patients from a single hospital or region[36], or included patients with many different causes of liver disease[13, 37]. This study also serves to augment prior research primarily conducted in Asia, thereby enhancing the global perspective.

During the first years of the pandemic, Sweden experienced high numbers of COVID-19 cases and higher mortality compared to neighbouring countries(38). Sweden implemented widespread testing measures, and all cases confirmed through polymerase chain reaction (PCR) were reported to a national communicable surveillance database SmiNet administered by the Public Health Agency of Sweden from 1 February 2020. Sweden also has unique register data for HBV patients, with advanced screening program, and most HBV-patients are followed by the Infectious disease clinics over time. This context offers a unique opportunity to study the impact on COVID-19 and HBV on a population level. Consequently, our study offers a clinically relevant assessment of the odds ratios pertaining to the development of clinically significant COVID-19 outcomes, on a national scale.

We acknowledge several limitations to our study. First, our research was conducted during the early phase of the pandemic when the alpha and beta variants of SARS-CoV-2 predominated in Sweden, and vaccination coverage was low[39]. In this period only elderly, or people with severe co-morbidities, were prioritized for vaccination, which is reflected in our univariable analysis where initially COVID-19 vaccination associated with severe COVID-19. However, following adjustment, the COVID-19 vaccine protected against severe COVID-19. Given the emergence of new variants and increased vaccination rates, the applicability of our findings to the current context may be limited. Second, identification of patients with cirrhosis/decompensation relied on diagnosis codes. This method identified only 98 individuals in the HBV with cirrhosis/decompensation group, and there is a possibility that some individuals meeting these criteria may not have received a formal diagnosis from their physicians. We also acknowledge the risk of missclassification, for example if patients with HBV infection have not been registered with a HBV diagnosis in any of the Swedish registers used in this study, which may affect the results. However, considering the strong associations in the results, the risk of underdiagnosed HBV infection is unlikely to have a major affect on the outcomes. Third, due to the utilization of national registers in our study, we lacked serological, laboratory and clinical data regarding liver function and HBV status, which could be of importance when interpreting the results.

In addition to the primary outcome, we analyzed several secondary outcomes, and outcomes stratified by cirrhosis and HBV. The reported p-values and confidence intervals were not adjusted for multiple testing. However, the results from the different analyses were in overall agreement.

## Conclusion

To our knowledge, this is the largest nationwide study, including all patients with laboratory-verified diagnosis of SARS-CoV-2 infection in a country, to identify the risk of severe COVID-19 in persons with chronic HBV infection. This study determines that chronic HBV infection gives a higher risk for severe outcomes of COVID-19, even after adjusting for the effect of important confounders. We also noted that individuals born in certain regions are more vulnerable than others. All these findings are important for future pandemics, and planning for vaccination strategies. These findings could change clinical practice and warrant a prioritisation of preventive and diagnostic strategies, which can affect treatment and, therefore, reduce the burden of morbidity and mortality in this patient group. However, caution should be exercised in generalizing these findings to the current context, given the evolving landscape of SARS-CoV-2 variants and increased COVID-19 vaccination coverage.

## Supplementary Information

Supplementary Material 1.

## Data Availability

No datasets were generated or analysed during the current study.

## Abbreviations

HBV: Hepatitis B virus
COVID-19: Coronavirus disease 2019
SARS-CoV2: Severe Acute Respiratory Syndrome Corona Virus 2
CDC: United States Centers for Disease Control and Prevention
ICU: Intensive Care Unit
HCC: Hepatocellular Carcinoma
LISA-registry: Longitudinal Integrated Database for Health Insurance and Labour Market Studies registry
ICD: International Statistical classification of Diseases
wCCI: weighted Charlson Comorbidity Index
OR: Odds ratio
SD: Standard Deviation
aOR: adjusted Odds Ratio
Non hosp: Non hospitalised
Hosp: Hospitalised
SES: socioeconomic status
PCR: Polymerase Chain Reaction
