# Supplementary material for: Hepatitis B associated with severe COVID-19: a nationwide cohort study in Sweden
Source: Virol J. 2025 Apr 30;22:127. doi: 10.1186/s12985-025-02743-5 (PMC12042447; doi:10.1186/s12985-025-02743-5)
Supplement: Supplementary file 1 — Supplementary Material 1. [file 12985_2025_2743_MOESM1_ESM.docx]

**Hepatitis B associated with severe COVID-19: A nationwide cohort study in Sweden.**

Journal: Virology Journal

Frida Jakobsson^1^, Osvaldo Fonseca-Rodríguez^1^, Hanna Jerndal^1^, Sebastian Kalucza^1^, Soo Aleman^2,3^, Marie Eriksson^4^, Anne-Marie Fors Connolly^1^*

1. Department of Clinical Microbiology, Umeå University, 90185 Umeå, Sweden

2. Department of Medicine, Huddinge Karolinska Institutet, 17177 Stockholm, Sweden

3. Department of Infectious Diseases, Karolinska University Hospital, 141 57 Stockholm, Sweden

4. Department of Statistics, Umeå School of Business, Economics and Statistics, Umeå University, SE 901 87 Umeå, Sweden.

* Correspondence to:

Anne-Marie Fors Connolly

Department of Clinical Microbiology

Umeå University

901 85 Sweden

Email: anne-marie.fors.connolly@umu.se

ORCID number: 0000-0001-9215-4047

**Additional files**

# Table 1. Diagnosis codes.

# Table 2. Secondary outcome. Death due to COVID-19.

# Table 3. Secondary outcome. Death by any cause.

# Table 4. Secondary outcome. Death by any cause. HBV-cohort divided in groups with or without cirrhosis.

# Table 5. Countries divided in regions according to ISO 3166

# **Table 1. Diagnosis codes.**

Chronic hepatitis B without cirrhosis: Diagnosis codes from group 1 given at any time.

Chronic hepatitis B with cirrhosis, with or without decompensation: Have diagnosis code B181E, B181G, OR any diagnosis codes in group 1 AND one or more of following diagnosis (=> belongs to group 2). Cirrhosis diagnosis at any given time. Decompensation diagnosis given >14 days before Covid-19 diagnosis date.

| **Diagnosis** | **ICD 9 (1987-1996)** | **ICD 10 (1997-to date)** |
| --- | --- | --- |
| Hepatitis B  (Group1) | 070D | B181  B181A  B181B  B181C  B181D  B181F  B181H  B181W  B181X |
| Cirrhosis  (Group 2) | 571X  571C  571F | B181E  B181G  K74  K746  K7460  K7469  K703 |
| Decompensation  (Group 2) | 570x  572C  572D  572E  572W  456A  348D  070C  070G  789F | K72  K720  K7200  K7201  K721  K7210  K7211  K729  K7290  K7291  R189  R18  I983  G934  G9340  G9341  G9349  G943  K767  I850  G929  B190  K704 |

# **Table 2. Secondary outcome. Death by COVID-19.**

# Death by covid vs non-hospitalized/hospitalized/ICU combined. Univariable and multivariable (adjusting for all other factors in the column) logistic regression modelling severe COVID-19 (Death). Exposure (Hepatitis B) in two categories (yes/no). Frequency (%), and odds ratios (OR) with 95% confidence intervals.

| **Dependent: Severity Death** |  | **Non-hosp·/Hosp/ICU** | **Dead** | **OR (univariable)** | **OR (multivariable)** |
| --- | --- | --- | --- | --- | --- |
| **Hepatitis B** | No hepatitis B | 836292 (98·6) | 11530 (1·4) | - | - |
|  | Hepatitis B | 2873 (99·0) | 29 (1·0) | 0·732 (0·496-1·035, p=0·095) | 1·168 (0·765-1·715, p=0·450) |
| **Age** | Mean (SD) | 43·8 (16·6) | 83·1 (9·9) | 1·147 (1·145-1·149, p<0·001) | 1·133 (1·131-1·136, p<0·001) |
| **Sex** | Women | 433583 (98·8) | 5266 (1·2) | - | - |
|  | Men | 405582 (98·5) | 6293 (1·5) | 1·278 (1·231-1·326, p<0·001) | 2·354 (2·250-2·462, p<0·001) |
| **Region of birth** | Northern Europe | 645459 (98·5) | 10074 (1·5) | - | - |
|  | Other Europe | 37119 (98·8) | 449 (1·2) | 0·775 (0·704-0·851, p<0·001) | 0·956 (0·856-1·066, p=0·424) |
|  | Africa | 17121 (99·4) | 110 (0·6) | 0·412 (0·339-0·494, p<0·001) | 1·335 (1·075-1·641, p=0·007) |
|  | Asia | 100738 (99·4) | 597 (0·6) | 0·380 (0·349-0·412, p<0·001) | 0·851 (0·768-0·942, p=0·002) |
|  | America | 11154 (99·3) | 81 (0·7) | 0·465 (0·371-0·575, p<0·001) | 0·965 (0·751-1·224, p=0·777) |
|  | Other/missing | 27574 (99·1) | 248 (0·9) | 0·576 (0·506-0·652, p<0·001) | 1·041 (0·901-1·198, p=0·577) |
| **COVID-19 vaccination** | 0 | 830242 (98·7) | 11272 (1·3) | - | - |
|  | 1 | 8923 (96·9) | 287 (3·1) | 2·369 (2·099-2·663, p<0·001) | 0·599 (0·524-0·681, p<0·001) |
| **wCCI group (excl liver disease)** | 0 | 789545 (99·2) | 6585 (0·8) | - | - |
|  | 1-2 | 28458 (94·2) | 1748 (5·8) | 7·365 (6·975-7·772, p<0·001) | 2·022 (1·896-2·156, p<0·001) |
|  | 3-4 | 6462 (80·8) | 1540 (19·2) | 28·574 (26·886-30·353, p<0·001) | 2·785 (2·590-2·993, p<0·001) |
|  | >=5 | 14700 (89·7) | 1686 (10·3) | 13·752 (13·000-14·539, p<0·001) | 3·012 (2·812-3·225, p<0·001) |
| **Education** | Tertiary | 323916 (99·4) | 1887 (0·6) | - | - |
|  | Secondary | 366704 (98·9) | 4214 (1·1) | 1·973 (1·868-2·083, p<0·001) | 1·200 (1·128-1·276, p<0·001) |
|  | Primary | 129108 (96·2) | 5032 (3·8) | 6·690 (6·344-7·058, p<0·001) | 1·253 (1·175-1·336, p<0·001) |
|  | Missing | 19437 (97·9) | 426 (2·1) | 3·762 (3·379-4·179, p<0·001) | 1·642 (1·428-1·887, p<0·001) |
| **Income** | Highest | 174801 (99·5) | 892 (0·5) | - | - |
|  | High | 175704 (99·6) | 680 (0·4) | 0·758 (0·686-0·838, p<0·001) | 1·088 (0·979-1·209, p=0·116) |
|  | Middle | 174092 (99·3) | 1220 (0·7) | 1·373 (1·260-1·498, p<0·001) | 1·480 (1·348-1·626, p<0·001) |
|  | Low | 166233 (97·4) | 4458 (2·6) | 5·255 (4·892-5·652, p<0·001) | 2·035 (1·876-2·209, p<0·001) |
|  | Lowest | 137646 (97·0) | 4293 (3·0) | 6·112 (5·688-6·575, p<0·001) | 2·372 (2·178-2·585, p<0·001) |
|  | Missing | 10689 (99·9) | 16 (0·1) | 0·293 (0·171-0·464, p<0·001) | 1·331 (0·755-2·180, p=0·288) |

ICU: intensive care unit admission. Non-hosp: Non-hospitalised. Hosp: Hospitalised. wCCI: weighted Charlson Comorbidity index.

# **Table 3. Secondary outcome. Death by any cause.**

Death by any cause vs. non-hospitalized/hospitalized/ICU combined. Univariable and multivariable (adjusting for all other factors in the column) logistic regression modelling. Death by any cause within 30 days from index date. 0=alive. 1=dead. Exposure (hepatitis B) in two categories (yes/no). Frequency (%), and odds ratios (OR) with 95% confidence intervals.

| **Dependent: Dead30d** |  | **0** | **1** | **OR (univariable)** | **OR (multivariable)** |
| --- | --- | --- | --- | --- | --- |
| **Hepatitis B** | No hepatitis B | 834167 (98.4) | 13655 (1.6) | - | - |
|  | Hepatitis B | 2870 (98.9) | 32 (1.1) | 0.681 (0.471-0.948, p=0.031) | 1.100 (0.735-1.589, p=0.627) |
| **Age (years)** | Mean (SD) | 43.7 (16.5) | 82.7 (10.4) | 1.148 (1.146-1.150, p<0.001) | 1.134 (1.132-1.136, p<0.001) |
| **Sex** | Women | 432562 (98.6) | 6287 (1.4) | - | - |
|  | Men | 404475 (98.2) | 7400 (1.8) | 1.259 (1.217-1.302, p<0.001) | 2.321 (2.225-2.420, p<0.001) |
| **Region of birth** | Northern Europe | 643561 (98.2) | 11972 (1.8) | - | - |
|  | Other Europe | 37023 (98.5) | 545 (1.5) | 0.791 (0.725-0.862, p<0.001) | 0.976 (0.881-1.079, p=0.638) |
|  | Africa | 17104 (99.3) | 127 (0.7) | 0.399 (0.333-0.473, p<0.001) | 1.256 (1.027-1.525, p=0.024) |
|  | Asia | 100673 (99.3) | 662 (0.7) | 0.353 (0.326-0.382, p<0.001) | 0.763 (0.692-0.840, p<0.001) |
|  | America | 11143 (99.2) | 92 (0.8) | 0.444 (0.359-0.542, p<0.001) | 0.890 (0.703-1.114, p=0.321) |
|  | Other/missing | 27533 (99.0) | 289 (1.0) | 0.564 (0.501-0.633, p<0.001) | 0.994 (0.869-1.134, p=0.934) |
| **COVID-19 Vaccination** | 0 | 828228 (98.4) | 13286 (1.6) | - | - |
|  | 1 | 8809 (95.6) | 401 (4.4) | 2.838 (2.560-3.136, p<0.001) | 0.705 (0.628-0.790, p<0.001) |
| **wCCI group** | 0 | 788041 (99.0) | 8089 (1.0) | - | - |
|  | 1-2 | 28242 (93.5) | 1964 (6.5) | 6.775 (6.438-7.126, p<0.001) | 1.889 (1.776-2.008, p<0.001) |
|  | 3-4 | 6314 (78.9) | 1688 (21.1) | 26.045 (24.572-27.594, p<0.001) | 2.559 (2.385-2.744, p<0.001) |
|  | >=5 | 14440 (88.1) | 1946 (11.9) | 13.129 (12.459-13.829, p<0.001) | 3.033 (2.840-3.238, p<0.001) |
| **Education** | Tertiary | 323532 (99.3) | 2271 (0.7) | - | - |
|  | Secondary | 365917 (98.7) | 5001 (1.3) | 1.947 (1.853-2.047, p<0.001) | 1.189 (1.123-1.260, p<0.001) |
|  | Primary | 128202 (95.6) | 5938 (4.4) | 6.599 (6.285-6.930, p<0.001) | 1.266 (1.193-1.344, p<0.001) |
|  | Missing | 19386 (97.6) | 477 (2.4) | 3.505 (3.169-3.869, p<0.001) | 1.615 (1.414-1.843, p<0.001) |
| **Income** | Highest | 174624 (99.4) | 1069 (0.6) | - | - |
|  | High | 175534 (99.5) | 850 (0.5) | 0.791 (0.723-0.866, p<0.001) | 1.145 (1.040-1.260, p=0.006) |
|  | Middle | 173827 (99.2) | 1485 (0.8) | 1.396 (1.290-1.510, p<0.001) | 1.544 (1.417-1.683, p<0.001) |
|  | Low | 165447 (96.9) | 5244 (3.1) | 5.178 (4.849-5.534, p<0.001) | 2.092 (1.941-2.257, p<0.001) |
|  | Lowest | 136920 (96.5) | 5019 (3.5) | 5.988 (5.606-6.402, p<0.001) | 2.454 (2.267-2.656, p<0.001) |
|  | Missing | 10685 (99.8) | 20 (0.2) | 0.306 (0.190-0.462, p<0.001) | 1.465 (0.883-2.294, p=0.115) |
|  |  |  |  |  |  |

ICU: intensive care unit admission. wCCI: weighted Charlson Comorbidity index.

# **Table 4. Secondary outcome. Death by any cause. HBV with or without cirrhosis.**

Univariable and multivariable (adjusting for all other factors in the column) logistic regression modelling. Death by any cause within 30 days from index date. 0=alive. 1=dead. Exposure (hepatitis B) in three categories (No hepatitis B, hepatitis B without cirrhosis, and Hepatitis B with cirrhosis). Frequency (%), and odds ratios (OR) with 95% confidence intervals.

| **Dependent: Dead 30d** |  | **0** | **1** | **OR (univariable)** | **OR (multivariable)** |
| --- | --- | --- | --- | --- | --- |
| **Hepatits B** | No hepatitis B | 834167 (98·4) | 13655 (1·6) | - | - |
|  | Hepatitis B without cirrhosis | 2781 (99·1) | 24 (0·9) | 0·527 (0·343-0·769, p=0·002) | 0·931 (0·584-1·414, p=0·750) |
|  | Hepatitis B with cirrhosis | 89 (91·8) | 8 (8·2) | 5·491 (2·450-10·620, p<0·001) | 2·354 (0·975-5·038, p=0·039) |
| **Age (years)** | Mean (SD) | 43·7 (16·5) | 82·7 (10·4) | 1·148 (1·146-1·150, p<0·001) | 1·134 (1·132-1·136, p<0·001) |
| **Sex** | Women | 432562 (98·6) | 6287 (1·4) | - | - |
|  | Men | 404475 (98·2) | 7400 (1·8) | 1·259 (1·217-1·302, p<0·001) | 2·320 (2·225-2·420, p<0·001) |
| **Region of birth** | Northern Europe | 643561 (98·2) | 11972 (1·8) | - | - |
|  | Other Europe | 37023 (98·5) | 545 (1·5) | 0·791 (0·725-0·862, p<0·001) | 0·976 (0·881-1·079, p=0·638) |
|  | Africa | 17104 (99·3) | 127 (0·7) | 0·399 (0·333-0·473, p<0·001) | 1·257 (1·027-1·526, p=0·023) |
|  | Asia | 100673 (99·3) | 662 (0·7) | 0·353 (0·326-0·382, p<0·001) | 0·763 (0·692-0·840, p<0·001) |
|  | America | 11143 (99·2) | 92 (0·8) | 0·444 (0·359-0·542, p<0·001) | 0·890 (0·703-1·114, p=0·322) |
|  | Other/missing | 27533 (99·0) | 289 (1·0) | 0·564 (0·501-0·633, p<0·001) | 0·994 (0·869-1·134, p=0·933) |
| **COVID-19 vaccination** | 0 | 828228 (98·4) | 13286 (1·6) | - | - |
|  | 1 | 8809 (95·6) | 401 (4·4) | 2·838 (2·560-3·136, p<0·001) | 0·705 (0·628-0·790, p<0·001) |
| **wCCI group** | 0 | 788041 (99·0) | 8089 (1·0) | - | - |
|  | 1-2 | 28242 (93·5) | 1964 (6·5) | 6·775 (6·438-7·126, p<0·001) | 1·889 (1·776-2·008, p<0·001) |
|  | 3-4 | 6314 (78·9) | 1688 (21·1) | 26·045 (24·572-27·594, p<0·001) | 2·558 (2·384-2·743, p<0·001) |
|  | >=5 | 14440 (88·1) | 1946 (11·9) | 13·129 (12·459-13·829, p<0·001) | 3·029 (2·836-3·233, p<0·001) |
| **Education** | Tertiary | 323532 (99·3) | 2271 (0·7) | - | - |
|  | Secondary | 365917 (98·7) | 5001 (1·3) | 1·947 (1·853-2·047, p<0·001) | 1·189 (1·123-1·259, p<0·001) |
|  | Primary | 128202 (95·6) | 5938 (4·4) | 6·599 (6·285-6·930, p<0·001) | 1·266 (1·193-1·343, p<0·001) |
|  | Missing | 19386 (97·6) | 477 (2·4) | 3·505 (3·169-3·869, p<0·001) | 1·614 (1·413-1·841, p<0·001) |
| **Income** | Highest | 174624 (99·4) | 1069 (0·6) | - | - |
|  | High | 175534 (99·5) | 850 (0·5) | 0·791 (0·723-0·866, p<0·001) | 1·145 (1·041-1·260, p=0·006) |
|  | Middle | 173827 (99·2) | 1485 (0·8) | 1·396 (1·290-1·510, p<0·001) | 1·544 (1·417-1·683, p<0·001) |
|  | Low | 165447 (96·9) | 5244 (3·1) | 5·178 (4·849-5·534, p<0·001) | 2·092 (1·941-2·257, p<0·001) |
|  | Lowest | 136920 (96·5) | 5019 (3·5) | 5·988 (5·606-6·402, p<0·001) | 2·454 (2·268-2·657, p<0·001) |
|  | Missing | 10685 (99·8) | 20 (0·2) | 0·306 (0·190-0·462, p<0·001) | 1·468 (0·885-2·298, p=0·113) |

ICU: intensive care unit admission. wCCI: weighted Charlson Comorbidity index.

**Table 5. Countries divided in regions according to ISO3166**

| **name** | **region** | **sub-region** | **intermediate-region** |
| --- | --- | --- | --- |
| Afghanistan | Asia | Southern Asia | |
| Åland Islands | Europe | Northern Europe | |
| Albania | Europe | Southern Europe | |
| Algeria | Africa | Northern Africa | |
| American Samoa | Oceania | Polynesia |  |
| Andorra | Europe | Southern Europe | |
| Angola | Africa | Sub-Saharan Africa | Middle Africa |
| Anguilla | Americas | Latin America and the Caribbean | Caribbean |
| Antarctica |  |  |  |
| Antigua and Barbuda | Americas | Latin America and the Caribbean | Caribbean |
| Argentina | Americas | Latin America and the Caribbean | South America |
| Armenia | Asia | Western Asia | |
| Aruba | Americas | Latin America and the Caribbean | Caribbean |
| Australia | Oceania | Australia and New Zealand | |
| Austria | Europe | Western Europe | |
| Azerbaijan | Asia | Western Asia | |
| Bahamas | Americas | Latin America and the Caribbean | Caribbean |
| Bahrain | Asia | Western Asia | |
| Bangladesh | Asia | Southern Asia | |
| Barbados | Americas | Latin America and the Caribbean | Caribbean |
| Belarus | Europe | Eastern Europe | |
| Belgium | Europe | Western Europe | |
| Belize | Americas | Latin America and the Caribbean | Central America |
| Benin | Africa | Sub-Saharan Africa | Western Africa |
| Bermuda | Americas | Northern America | |
| Bhutan | Asia | Southern Asia | |
| Bolivia (Plurinational State of) | Americas | Latin America and the Caribbean | South America |
| Bonaire, Sint Eustatius and Saba | Americas | Latin America and the Caribbean | Caribbean |
| Bosnia and Herzegovina | Europe | Southern Europe | |
| Botswana | Africa | Sub-Saharan Africa | Southern Africa |
| Bouvet Island | Americas | Latin America and the Caribbean | South America |
| Brazil | Americas | Latin America and the Caribbean | South America |
| British Indian Ocean Territory | Africa | Sub-Saharan Africa | Eastern Africa |
| Brunei Darussalam | Asia | South-eastern Asia | |
| Bulgaria | Europe | Eastern Europe | |
| Burkina Faso | Africa | Sub-Saharan Africa | Western Africa |
| Burundi | Africa | Sub-Saharan Africa | Eastern Africa |
| Cabo Verde | Africa | Sub-Saharan Africa | Western Africa |
| Cambodia | Asia | South-eastern Asia | |
| Cameroon | Africa | Sub-Saharan Africa | Middle Africa |
| Canada | Americas | Northern America | |
| Cayman Islands | Americas | Latin America and the Caribbean | Caribbean |
| Central African Republic | Africa | Sub-Saharan Africa | Middle Africa |
| Chad | Africa | Sub-Saharan Africa | Middle Africa |
| Chile | Americas | Latin America and the Caribbean | South America |
| China | Asia | Eastern Asia | |
| Christmas Island | Oceania | Australia and New Zealand | |
| Cocos (Keeling) Islands | Oceania | Australia and New Zealand | |
| Colombia | Americas | Latin America and the Caribbean | South America |
| Comoros | Africa | Sub-Saharan Africa | Eastern Africa |
| Congo | Africa | Sub-Saharan Africa | Middle Africa |
| Congo, Democratic Republic of the | Africa | Sub-Saharan Africa | Middle Africa |
| Cook Islands | Oceania | Polynesia |  |
| Costa Rica | Americas | Latin America and the Caribbean | Central America |
| Côte d'Ivoire | Africa | Sub-Saharan Africa | Western Africa |
| Croatia | Europe | Southern Europe | |
| Cuba | Americas | Latin America and the Caribbean | Caribbean |
| Curaçao | Americas | Latin America and the Caribbean | Caribbean |
| Cyprus | Asia | Western Asia | |
| Czechia | Europe | Eastern Europe | |
| Denmark | Europe | Northern Europe | |
| Djibouti | Africa | Sub-Saharan Africa | Eastern Africa |
| Dominica | Americas | Latin America and the Caribbean | Caribbean |
| Dominican Republic | Americas | Latin America and the Caribbean | Caribbean |
| Ecuador | Americas | Latin America and the Caribbean | South America |
| Egypt | Africa | Northern Africa | |
| El Salvador | Americas | Latin America and the Caribbean | Central America |
| Equatorial Guinea | Africa | Sub-Saharan Africa | Middle Africa |
| Eritrea | Africa | Sub-Saharan Africa | Eastern Africa |
| Estonia | Europe | Northern Europe | |
| Eswatini | Africa | Sub-Saharan Africa | Southern Africa |
| Ethiopia | Africa | Sub-Saharan Africa | Eastern Africa |
| Falkland Islands (Malvinas) | Americas | Latin America and the Caribbean | South America |
| Faroe Islands | Europe | Northern Europe | |
| Fiji | Oceania | Melanesia |  |
| Finland | Europe | Northern Europe | |
| France | Europe | Western Europe | |
| French Guiana | Americas | Latin America and the Caribbean | South America |
| French Polynesia | Oceania | Polynesia |  |
| French Southern Territories | Africa | Sub-Saharan Africa | Eastern Africa |
| Gabon | Africa | Sub-Saharan Africa | Middle Africa |
| Gambia | Africa | Sub-Saharan Africa | Western Africa |
| Georgia | Asia | Western Asia | |
| Germany | Europe | Western Europe | |
| Ghana | Africa | Sub-Saharan Africa | Western Africa |
| Gibraltar | Europe | Southern Europe | |
| Greece | Europe | Southern Europe | |
| Greenland | Americas | Northern America | |
| Grenada | Americas | Latin America and the Caribbean | Caribbean |
| Guadeloupe | Americas | Latin America and the Caribbean | Caribbean |
| Guam | Oceania | Micronesia |  |
| Guatemala | Americas | Latin America and the Caribbean | Central America |
| Guernsey | Europe | Northern Europe | Channel Islands |
| Guinea | Africa | Sub-Saharan Africa | Western Africa |
| Guinea-Bissau | Africa | Sub-Saharan Africa | Western Africa |
| Guyana | Americas | Latin America and the Caribbean | South America |
| Haiti | Americas | Latin America and the Caribbean | Caribbean |
| Heard Island and McDonald Islands | Oceania | Australia and New Zealand | |
| Holy See | Europe | Southern Europe | |
| Honduras | Americas | Latin America and the Caribbean | Central America |
| Hong Kong | Asia | Eastern Asia | |
| Hungary | Europe | Eastern Europe | |
| Iceland | Europe | Northern Europe | |
| India | Asia | Southern Asia | |
| Indonesia | Asia | South-eastern Asia | |
| Iran (Islamic Republic of) | Asia | Southern Asia | |
| Iraq | Asia | Western Asia | |
| Ireland | Europe | Northern Europe | |
| Isle of Man | Europe | Northern Europe | |
| Israel | Asia | Western Asia | |
| Italy | Europe | Southern Europe | |
| Jamaica | Americas | Latin America and the Caribbean | Caribbean |
| Japan | Asia | Eastern Asia | |
| Jersey | Europe | Northern Europe | Channel Islands |
| Jordan | Asia | Western Asia | |
| Kazakhstan | Asia | Central Asia | |
| Kenya | Africa | Sub-Saharan Africa | Eastern Africa |
| Kiribati | Oceania | Micronesia |  |
| Korea (Democratic People's Republic of) | Asia | Eastern Asia | |
| Korea, Republic of | Asia | Eastern Asia | |
| Kuwait | Asia | Western Asia | |
| Kyrgyzstan | Asia | Central Asia | |
| Lao People's Democratic Republic | Asia | South-eastern Asia | |
| Latvia | Europe | Northern Europe | |
| Lebanon | Asia | Western Asia | |
| Lesotho | Africa | Sub-Saharan Africa | Southern Africa |
| Liberia | Africa | Sub-Saharan Africa | Western Africa |
| Libya | Africa | Northern Africa | |
| Liechtenstein | Europe | Western Europe | |
| Lithuania | Europe | Northern Europe | |
| Luxembourg | Europe | Western Europe | |
| Macao | Asia | Eastern Asia | |
| Madagascar | Africa | Sub-Saharan Africa | Eastern Africa |
| Malawi | Africa | Sub-Saharan Africa | Eastern Africa |
| Malaysia | Asia | South-eastern Asia | |
| Maldives | Asia | Southern Asia | |
| Mali | Africa | Sub-Saharan Africa | Western Africa |
| Malta | Europe | Southern Europe | |
| Marshall Islands | Oceania | Micronesia |  |
| Martinique | Americas | Latin America and the Caribbean | Caribbean |
| Mauritania | Africa | Sub-Saharan Africa | Western Africa |
| Mauritius | Africa | Sub-Saharan Africa | Eastern Africa |
| Mayotte | Africa | Sub-Saharan Africa | Eastern Africa |
| Mexico | Americas | Latin America and the Caribbean | Central America |
| Micronesia (Federated States of) | Oceania | Micronesia |  |
| Moldova, Republic of | Europe | Eastern Europe | |
| Monaco | Europe | Western Europe | |
| Mongolia | Asia | Eastern Asia | |
| Montenegro | Europe | Southern Europe | |
| Montserrat | Americas | Latin America and the Caribbean | Caribbean |
| Morocco | Africa | Northern Africa | |
| Mozambique | Africa | Sub-Saharan Africa | Eastern Africa |
| Myanmar | Asia | South-eastern Asia | |
| Namibia | Africa | Sub-Saharan Africa | Southern Africa |
| Nauru | Oceania | Micronesia |  |
| Nepal | Asia | Southern Asia | |
| Netherlands | Europe | Western Europe | |
| New Caledonia | Oceania | Melanesia |  |
| New Zealand | Oceania | Australia and New Zealand | |
| Nicaragua | Americas | Latin America and the Caribbean | Central America |
| Niger | Africa | Sub-Saharan Africa | Western Africa |
| Nigeria | Africa | Sub-Saharan Africa | Western Africa |
| Niue | Oceania | Polynesia |  |
| Norfolk Island | Oceania | Australia and New Zealand | |
| North Macedonia | Europe | Southern Europe | |
| Northern Mariana Islands | Oceania | Micronesia |  |
| Norway | Europe | Northern Europe | |
| Oman | Asia | Western Asia | |
| Pakistan | Asia | Southern Asia | |
| Palau | Oceania | Micronesia |  |
| Palestine, State of | Asia | Western Asia | |
| Panama | Americas | Latin America and the Caribbean | Central America |
| Papua New Guinea | Oceania | Melanesia |  |
| Paraguay | Americas | Latin America and the Caribbean | South America |
| Peru | Americas | Latin America and the Caribbean | South America |
| Philippines | Asia | South-eastern Asia | |
| Pitcairn | Oceania | Polynesia |  |
| Poland | Europe | Eastern Europe | |
| Portugal | Europe | Southern Europe | |
| Puerto Rico | Americas | Latin America and the Caribbean | Caribbean |
| Qatar | Asia | Western Asia | |
| Réunion | Africa | Sub-Saharan Africa | Eastern Africa |
| Romania | Europe | Eastern Europe | |
| Russian Federation | Europe | Eastern Europe | |
| Rwanda | Africa | Sub-Saharan Africa | Eastern Africa |
| Saint Barthélemy | Americas | Latin America and the Caribbean | Caribbean |
| Saint Helena, Ascension and Tristan da Cunha | Africa | Sub-Saharan Africa | Western Africa |
| Saint Kitts and Nevis | Americas | Latin America and the Caribbean | Caribbean |
| Saint Lucia | Americas | Latin America and the Caribbean | Caribbean |
| Saint Martin (French part) | Americas | Latin America and the Caribbean | Caribbean |
| Saint Pierre and Miquelon | Americas | Northern America | |
| Saint Vincent and the Grenadines | Americas | Latin America and the Caribbean | Caribbean |
| Samoa | Oceania | Polynesia |  |
| San Marino | Europe | Southern Europe | |
| Sao Tome and Principe | Africa | Sub-Saharan Africa | Middle Africa |
| Saudi Arabia | Asia | Western Asia | |
| Senegal | Africa | Sub-Saharan Africa | Western Africa |
| Serbia | Europe | Southern Europe | |
| Seychelles | Africa | Sub-Saharan Africa | Eastern Africa |
| Sierra Leone | Africa | Sub-Saharan Africa | Western Africa |
| Singapore | Asia | South-eastern Asia | |
| Sint Maarten (Dutch part) | Americas | Latin America and the Caribbean | Caribbean |
| Slovakia | Europe | Eastern Europe | |
| Slovenia | Europe | Southern Europe | |
| Solomon Islands | Oceania | Melanesia |  |
| Somalia | Africa | Sub-Saharan Africa | Eastern Africa |
| South Africa | Africa | Sub-Saharan Africa | Southern Africa |
| South Georgia and the South Sandwich Islands | Americas | Latin America and the Caribbean | South America |
| South Sudan | Africa | Sub-Saharan Africa | Eastern Africa |
| Spain | Europe | Southern Europe | |
| Sri Lanka | Asia | Southern Asia | |
| Sudan | Africa | Northern Africa | |
| Suriname | Americas | Latin America and the Caribbean | South America |
| Svalbard and Jan Mayen | Europe | Northern Europe | |
| Sweden | Europe | Northern Europe | |
| Switzerland | Europe | Western Europe | |
| Syrian Arab Republic | Asia | Western Asia | |
| Taiwan, Province of China | Asia | Eastern Asia | |
| Tajikistan | Asia | Central Asia | |
| Tanzania, United Republic of | Africa | Sub-Saharan Africa | Eastern Africa |
| Thailand | Asia | South-eastern Asia | |
| Timor-Leste | Asia | South-eastern Asia | |
| Togo | Africa | Sub-Saharan Africa | Western Africa |
| Tokelau | Oceania | Polynesia |  |
| Tonga | Oceania | Polynesia |  |
| Trinidad and Tobago | Americas | Latin America and the Caribbean | Caribbean |
| Tunisia | Africa | Northern Africa | |
| Turkey | Asia | Western Asia | |
| Turkmenistan | Asia | Central Asia | |
| Turks and Caicos Islands | Americas | Latin America and the Caribbean | Caribbean |
| Tuvalu | Oceania | Polynesia |  |
| Uganda | Africa | Sub-Saharan Africa | Eastern Africa |
| Ukraine | Europe | Eastern Europe | |
| United Arab Emirates | Asia | Western Asia | |
| United Kingdom of Great Britain and Northern Ireland | Europe | Northern Europe | |
| United States of America | Americas | Northern America | |
| United States Minor Outlying Islands | Oceania | Micronesia |  |
| Uruguay | Americas | Latin America and the Caribbean | South America |
| Uzbekistan | Asia | Central Asia | |
| Vanuatu | Oceania | Melanesia |  |
| Venezuela (Bolivarian Republic of) | Americas | Latin America and the Caribbean | South America |
| Viet Nam | Asia | South-eastern Asia | |
| Virgin Islands (British) | Americas | Latin America and the Caribbean | Caribbean |
| Virgin Islands (U.S.) | Americas | Latin America and the Caribbean | Caribbean |
| Wallis and Futuna | Oceania | Polynesia |  |
| Western Sahara | Africa | Northern Africa | |
| Yemen | Asia | Western Asia | |
| Zambia | Africa | Sub-Saharan Africa | Eastern Africa |
| Zimbabwe | Africa | Sub-Saharan Africa | Eastern Africa |
